# Supplementary material for: The yielding transition in amorphous solids under oscillatory shear deformation
Source: Nat Commun. 2017 Mar 1;8:14653. doi: 10.1038/ncomms14653 (PMC5337995; doi:10.1038/ncomms14653)
Supplement: Supplementary Information — Supplementary Figures and Supplementary References [file ncomms14653-s1.pdf]

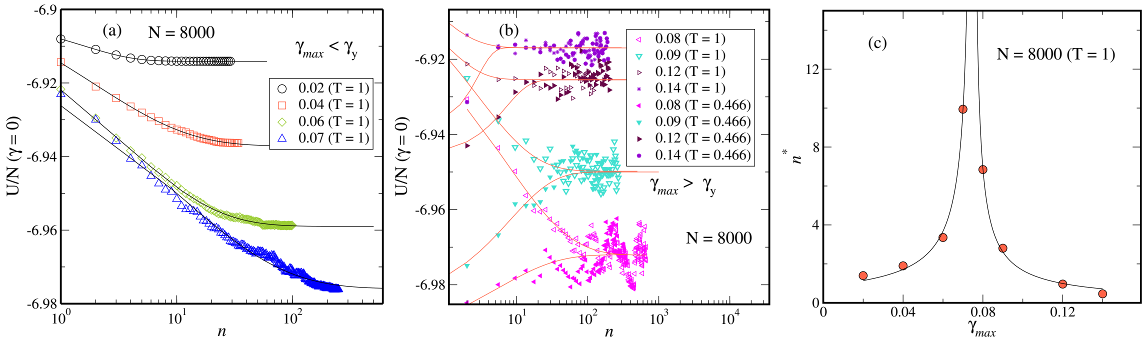

Supplementary Figure 1: **Approach to the steady state.** Energy vs. cycle for different strain amplitudes **(a)** below and **(b)** above the transition. **(c)** The decay constant n^* to reach the steady state. We analyse the potential energy $U(\gamma = 0)$ *vs.* the number of cycles n to probe the approach to the steady state, and denote by n^* the indicative number of cycles needed to reach the steady state, and is obtained as a decay constant by fitting the cycle dependence of energies to the form $U(n) = U_\infty + \Delta U_0 \exp\left[-(n/n^*)^\beta\right]$. These fits are shown in panels (a), (b) for $\gamma_{max} < \gamma_y$ (for the $T = 1$ cases) and $\gamma_{max} > \gamma_y$ (for both $T = 1$ and $T = 0.466$) respectively. Panel (c) shows that n^* grows strongly on approaching the yield value of γ_{max} between 0.07 and 0.08. The fit lines are guides to the eye.

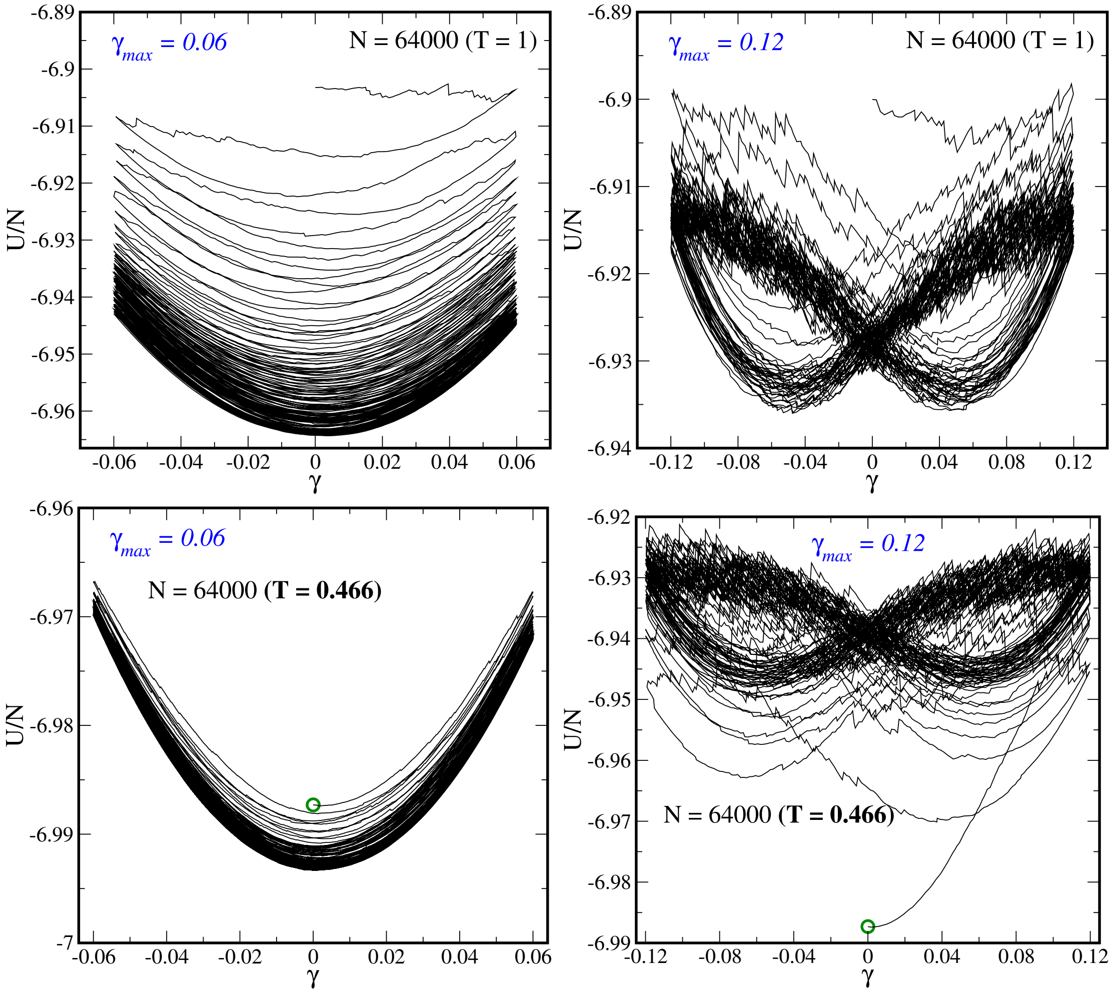

Supplementary Figure 2: **Evolution of energy with cycles.** Energy *vs.* strain over cycles starting with the undeformed glass, showing (for $\gamma_{max} = 0.06$) the approach to a single minimum at $\gamma = 0$, and bifurcation into two minima (for $\gamma_{max} = 0.12$) at finite strain. Data are shown for both well annealed ($T = 0.466$) and poorly annealed ($T = 1.0$) glasses.

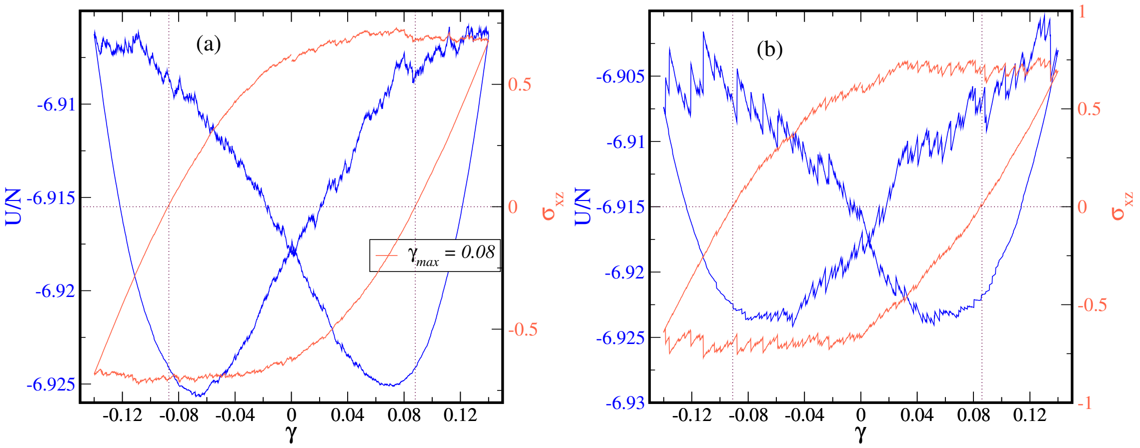

Supplementary Figure 3: **Stress and energy** *vs.* **strain in the steady state.** Stress *vs.* strain (red lines) and energy *vs.* strain (blue lines) are shown, along with with dotted lines as guides to the eye, to locate $\sigma_{xz} = 0$. Data shown are **(a)** averaged over 10 cycles and **(b)** for a single cycle, in the steady state. The strain at $U = U_{min}$ and $\sigma_{xz} = 0$ are represented by $\gamma_{U_{min}}$ and γ_{σ_0}. To test if the location of the energy minima coincide with the strain at zero stress, *i. e.* if $\gamma_{U_{min}} = \gamma_{\sigma_0}$, we plot the energy and the stress loops for $\gamma_{max} = 0.14$ of N = 64000 (T=1). Though their values are close, we find $\gamma_{U_{min}} \neq \gamma_{\sigma_0}$ (for $\gamma_{max} > \gamma_y$).

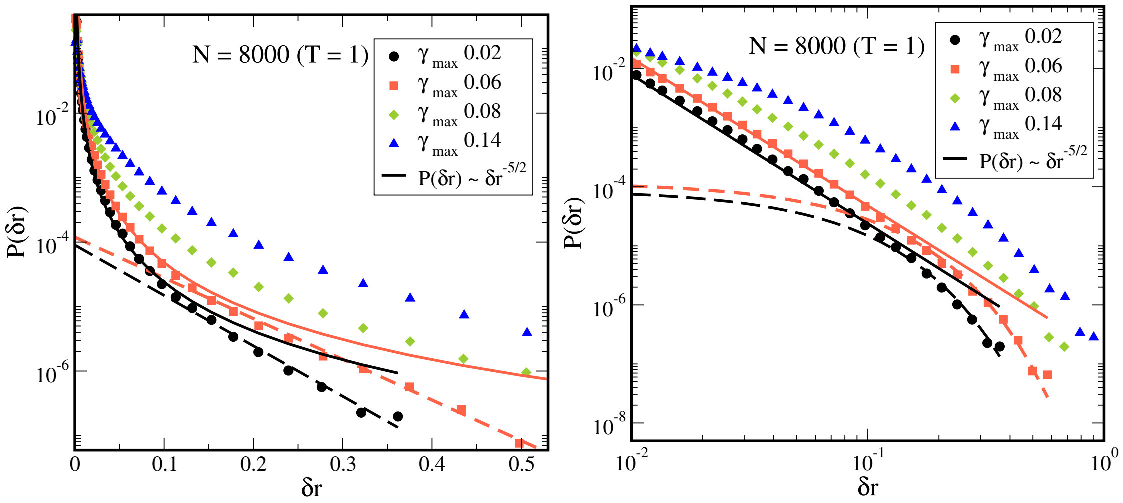

Supplementary Figure 4: **Identification of particles undergoing plastic displacements.** Distributions of the particle displacements at the plastic events shown in semi-log and log-log scales for various strain amplitudes. Here we describe how particles that are labeled "active", that undergo plastic deformation during an energy drop, are identified, based on previous work [see supplementary references 1, 2]. In the presence of a plastic rearrangement, it is found that the distribution of single particle displacements $p(\delta r)$ displays an exponential tail, corresponding to plastic displacements, and a power law distribution at smaller values with an exponent of $-5/2$ which may be deduced from assuming that the rest of the system undergoes an elastic deformation owing to the stresses created by the plastic deformation. Such an expectation is clearly satisfied at low strain amplitudes γ_{max}, but (a) the location of the crossover depends on the strain amplitude, and (b) the distinction becomes less clear at large strain amplitudes. We wish to include all particles that take part in plastic deformation, but to exclude those undergoing elastic displacements. As a conservative choice of cutoff, we use the cutoff that is clear and applicable for the case of $\gamma_{max} = 0.02$, namely $\delta r = 0.1$.

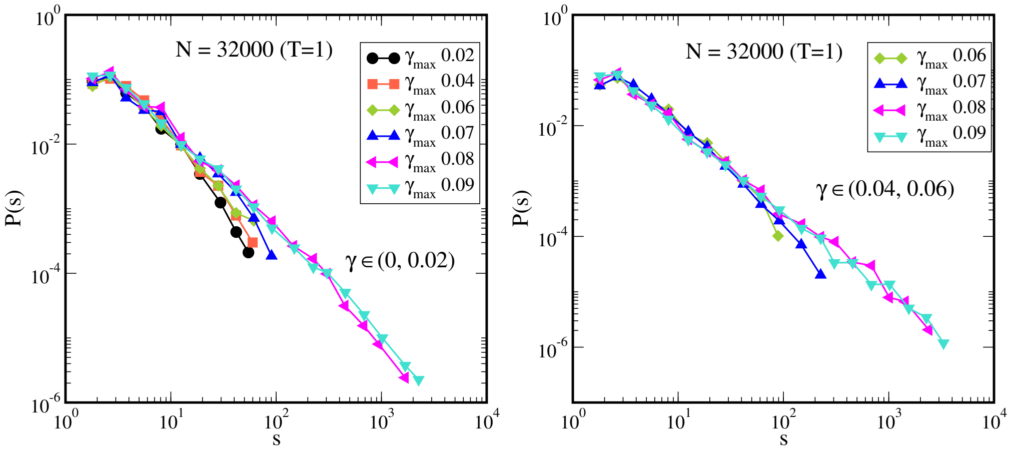

Supplementary Figure 5: **Avalanche distributions in different strain windows.** Distributions of avalanche sizes within specified windows of strain [(left panel) 0 to 0.02, and (right panel) 0.04 to 0.06] for different strain amplitudes. We show here the distribution of avalanche sizes that result when specific bins in the strain γ are considered, for different amplitudes γ_{max}, with results averaged over the first quadrant of cycles of strain. The figure shows the distribution of avalanche sizes in the strain window of (a) 0 to 0.02, and (b) 0.04 to 0.06, for different values of γ_{max} for which we sample the strain window in the course of a full cycle. We note that in both cases, the distributions fall into two categories, one with $\gamma < \gamma_y$ and the other with $\gamma > \gamma_y$. In each category, the distributions are largely independent of the value of γ_{max}, but the distributions for the two categories are distinct.

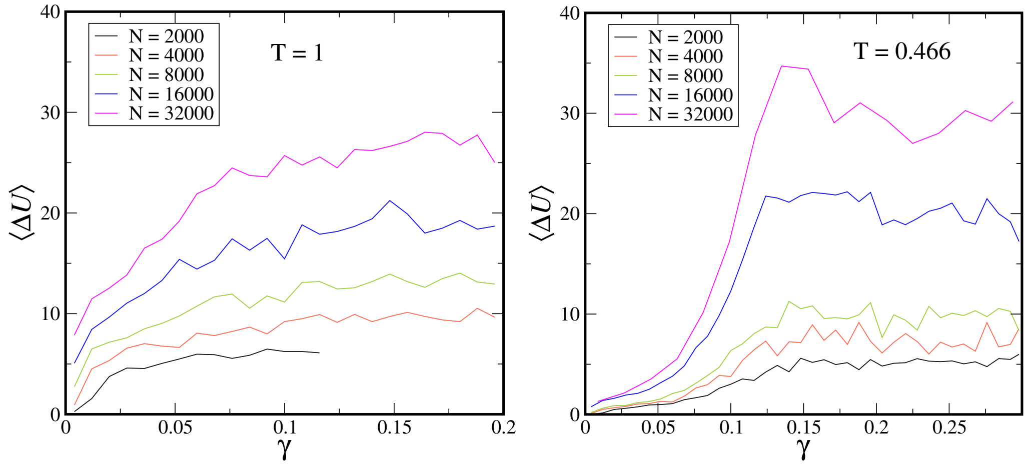

Supplementary Figure 6: **Energy drops for uniform strain.** Mean energy drops *vs.* strain for various system sizes for uniform strain. We present the mean energy drop for uniform strain, for $T = 1$ and $T = 0.466$, for a range of system sizes for a range of system sizes. We see that the mean energy drops for the two temperatures are significantly different, but in each case show similar trends in their system size dependence.

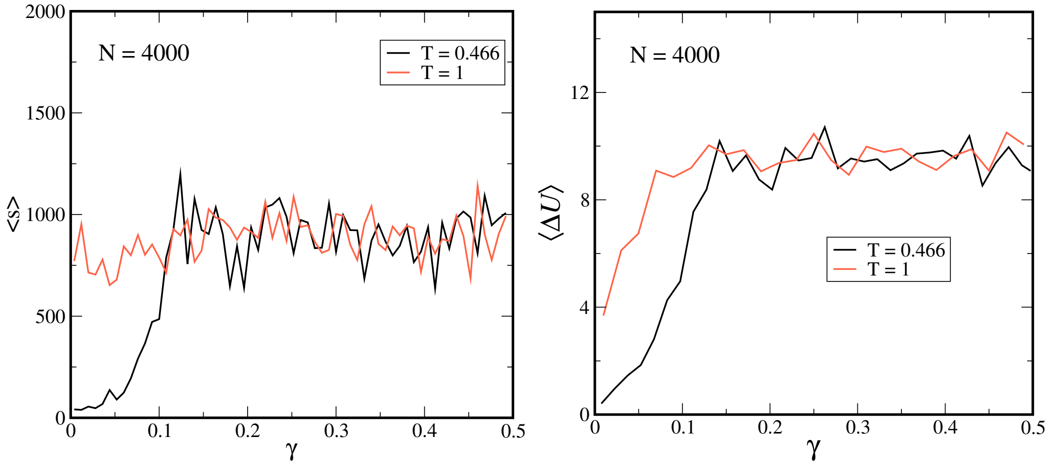

Supplementary Figure 7: **Comparison of means cluster size and energy drops for differently annealed glasses under uniform strain.** Mean avalanche size and mean energy drops *vs.* strain for $T = 1$ and $T = 0.466$, $N = 4000$. The behaviour for the two cases is very different at strains below the yield strain, thus making it difficult to provide a general characterisation of the avalanches below the yield strain identified by oscillatory deformation. Further, we note that for $T = 1$, the energy drops and avalanche sizes below the yield strain remain high and comparable to values above yield strain.

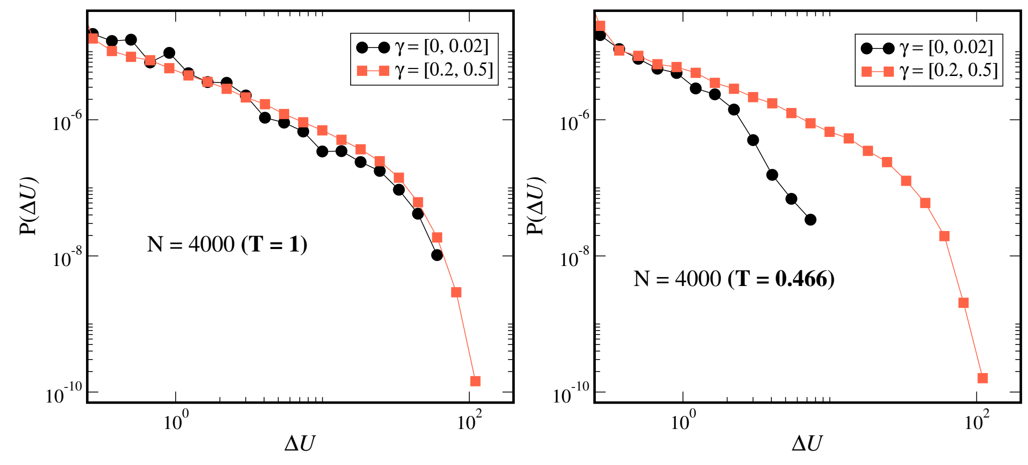

Supplementary Figure 8: **Distribution of energy drops under uniform strain.** Distributions of energy drops for two windows of strain, below and above the yielding transition, shown for $T = 1$ and $T = 0.466$. The figure shows the distributions of energy drops for two strain intervals (one below, $\{0, 0.02\}$, and one above, $\{0.2, 0.5\}$, the yield strian). For $T = 1$, the two distributions do not differ, whereas for $T = 0.466$, they are widely separated.

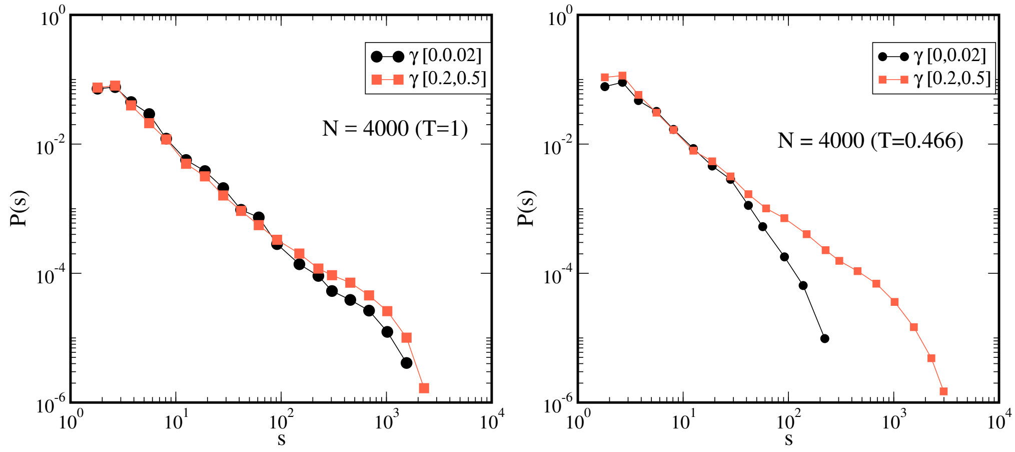

Supplementary Figure 9: **Distribution of avalanche sizes under uniform strain.** Distributions of avalanche sizes for two windows of strain, below and above the yielding transition, shown for $T = 1$ and $T = 0.466$.

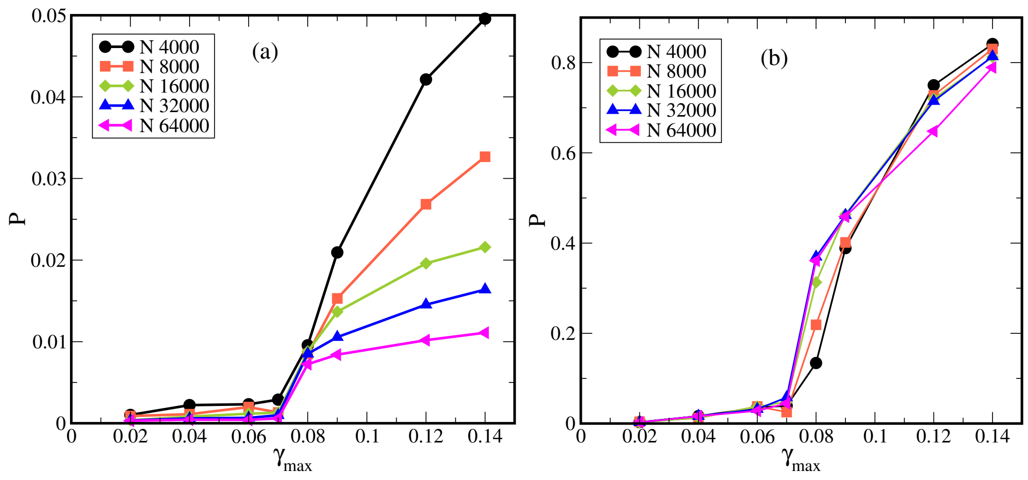

Supplementary Figure 10: **Fraction of active particles** *vs.* **strain amplitude and system size.** Fraction of active particles P *vs.* strain amplitude γ_{max}, for different system sizes, **(a)** for individual drop events, and **(b)** accumulated over the first quadrant of cycles of strain. The averages are performed over the first quadrant of the strain cycles. In panel **(a)** the probability P is for individual drop events, averaged over all events, whereas in panel **(b)** P is obtained for each cycle by accumulating all particles that are active in any of the drop events that occur, and the averaging is done over all cycles. In both cases, P changes sharply across the yield strain, but with different system size dependence.

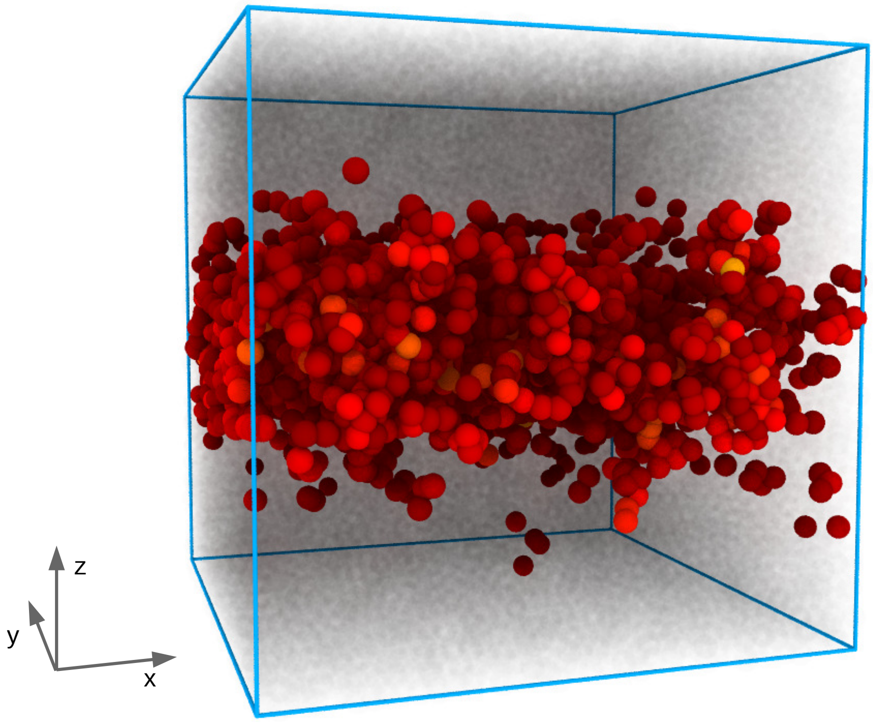

Supplementary Figure 11: **Shear banding.** Shear bands formed by particles that have scalar displacements greater than $0.6\sigma_{AA}$ at the completion of a cycle in the steady states (shown in red), for strain amplitude $\gamma_{max} = 0.08$ for $N = 64000$, $T = 1$. Above the yielding strain amplitude, particles that undergo large displacements form shear bands. The thickness of the shear bands increases with the strain amplitude, as will be discussed further elsewhere.

Supplementary References

1. Fiocco, D., Foffi, G. and Sastry, S. Encoding of memory in sheared amorphous solids. *Phys. Rev. Lett.* **112**, 025702 (2014).

2. Schrøder,T. B., Sastry, S., Dyre, J. C., and Glotzer, S. C., Crossover to potential energy landscape dominated dynamics in a model glass-forming liquid *J. Chem. Phys.* **112**, 9834 (2000).
